# Protein docking by Rotation-Based Uniform Sampling (RotBUS) with fast computing of intermolecular contact distance and residue desolvation

**DOI:** 10.1186/1471-2105-11-352

**Published:** 2010-06-28

**Authors:** Albert Solernou, Juan Fernandez-Recio

**Affiliations:** 1 Life Sciences Department, Barcelona Supercomputing Center, Barcelona, Spain

## Abstract

**Background:**

Protein-protein interactions are fundamental for the majority of cellular processes and their study is of enormous biotechnological and therapeutic interest. In recent years, a variety of computational approaches to the protein-protein docking problem have been reported, with encouraging results. Most of the currently available protein-protein docking algorithms are composed of two clearly defined parts: the sampling of the rotational and translational space of the interacting molecules, and the scoring and clustering of the resulting orientations. Although this kind of strategy has shown some of the most successful results in the CAPRI blind test http://www.ebi.ac.uk/msd-srv/capri, more efforts need to be applied. Thus, the sampling protocol should generate a pool of conformations that include a sufficient number of near-native ones, while the scoring function should discriminate between near-native and non-near-native proposed conformations. On the other hand, protocols to efficiently include full flexibility on the protein structures are increasingly needed.

**Results:**

In these work we present new computational tools for protein-protein docking. We describe here the RotBUS (Rotation-Based Uniform Sampling) method to generate uniformly distributed sets of rigid-body docking poses, with a new fast calculation of the optimal contacting distance between molecules. We have tested the method on a standard benchmark of unbound structures and we can find near-native solutions in 100% of the cases. After applying a new fast filtering scheme based on residue-based desolvation, in combination with FTDock plus pyDock scoring, near-native solutions are found with rank ≤ 50 in 39% of the cases. Knowledge-based experimental restraints can be easily included to reduce computational times during sampling and improve success rates, and the method can be extended in the future to include flexibility of the side-chains.

**Conclusions:**

This new sampling algorithm has the advantage of its high speed achieved by fast computing of the intermolecular distance based on a coarse representation of the interacting surfaces. In addition, a fast desolvation scoring permits the screening of millions of conformations at low computational cost, without compromising accuracy. The protocol presented here can be used as a framework to include restraints, flexibility and ensemble docking approaches.

## Background

Protein-protein interactions are essential for living organisms. They are involved in most of the key biological and biochemical processes, such as signal transduction, redox reactions, immune response and protein transport, among many others. Thus, understanding protein-protein association is the object of an increasing interest, not only from a basic physico-chemical point of view, but also for biotechnological and therapeutic reasons, with promising applications for drug design. However, experimental data on complex formation is scarce. Although in recent years techniques like NMR or X-ray crystallography have fuelled the field of structural biology, the number of known protein-protein complex structures remains low. Therefore, the prediction of protein-protein interactions have become one of the most active and creative fields in computational physico-chemistry and biology. Strictly the native complex consists of an ensemble of structures belonging to the free energy minimum of the system formed by the receptor, the ligand, and the water and ions surrounding them. Thus, in principle, using molecular simulations via molecular dynamics or Monte Carlo strategies, one should calculate the free energy of the system, and discriminate between native and non native conformations (for a recent review of free energy calculations we refer the reader to [1]). However, the amount of calculation that must be carried in a system with a pair of medium-sized proteins is prohibitive. In this way, docking strategies follow two main simplifications. On the one hand the native state of the complex is not seen as the ensemble of structures referred below, but as a single structure. On the other hand, free energy calculations are replaced by a scoring function which should distinguish the native structures in a pool of conformations.

Most of the docking algorithms are clearly divided in two fundamental parts: the search of conformations, and the scoring function. The first stage should generate a pool of conformations with a sufficient number of them similar to the native complex; the latter should discriminate between the near-native and non-near-native proposed conformations. One of the limitations of current docking simulations is precisely the conformational search phase, which is not able to produce sufficient number of near-native docking solutions within the docking pool that can be correctly identified in subsequent scoring or refinement steps.

There are many available search methods, but most of them can be classified in two basic types: systematic and heuristic. On the one side, systematic sampling methods try to explore the whole or a subspace of the available conformations of the system. The large number of degrees of freedom makes unfeasible a complete conformational search in a reasonable time, so most of the reported docking algorithms treat both proteins of the complex as rigid bodies. This dramatic approximation has worked reasonably well for a significant number of protein-protein complexes, especially those with neither hinge-motions nor disorder-order transitions and where the conformational changes are limited to the side-chains. On the basis of this approach, protein docking has benefited from Fast Fourier Transform (FFT) [2-7], Spherical Polar Correlations [8] or Geometric-Hashing [9] algorithms for a fast search of the position of the interacting molecules, so these have always been the most popular docking methods. However, challenging cases involve major conformational changes upon binding, in which rigid-body docking methods often struggle to find any reasonable solution. Another major limitation is that these methods have difficulties in including sophisticated scoring functions to evaluate the docking orientations and thus are mostly based on geometrical criteria such as surface complementarity. A last (but not least) problem is that, in most of FFT-based methods, one monomer is fixed while the other is translated and rotated. This sampling strategy is obviously not the one that uses nature to face up the interacting proteins, but was chosen for practical issues of implementation. However, as recently reported [10], other sampling techniques based on orientational changes of both molecules can provide a more natural sampling and lead to an improvement of computational efficiency.

On the other side, heuristic searching methods do not need to exhaustively sample the whole conformational space, which presents some advantages, such as being able to include flexibility during the conformational search and use more sophisticated scoring functions. For instance, the ICM-DISCO docking method was based on a Monte-Carlo search of the ligand molecule using the binding potentials pre-calculated in a 3 D grid for the receptor, followed by the optimisation of the ligand interface side-chains in the torsional space [11]. These methods can provide more accurate geometries, but they are usually much slower than the systematic FFT-based search approaches.

In this paper we will try to overcome the limitations of current docking search methods, and propose an algorithm to uniformly generate docking orientations for a two rigid body system in a fast manner, with potential inclusion of flexibility. The method, to which we refer as RotBUS (Rotation Based Uniform Sampling) generates orientations in such a way that subspaces can be easily explored, which would allow to combine the speed of FFT-based systematic approaches with the traditional advantages of heuristic methods with no extra cost. The method has been applied to an 84 complex benchmark set [12], widely used to evaluate docking algorithms. After filtering the generated conformations with a residue-based solvation potential, they are scored with atom-based pyDock scoring function [13].

## Methods

We describe here new procedures to generate different configurations in a two-protein system, based on uniform sampling of the rotational space of the two interacting proteins and fast calculation of their optimal distance. The two proteins are considered as rigid bodies. In this work, the larger protein is defined as the receptor, and the smaller one the ligand. First we will define uniform distributions of points over the spheres around the interacting molecules. This will be used later to uniformly sample orientations by three different methods, inspired by early work on uniform random rotations [14]. Finally, for each orientation the optimal distance between these molecules will be calculated.

### Uniform sampling of molecular orientations

#### Uniform distribution of points around the molecule

**Figure 1:**
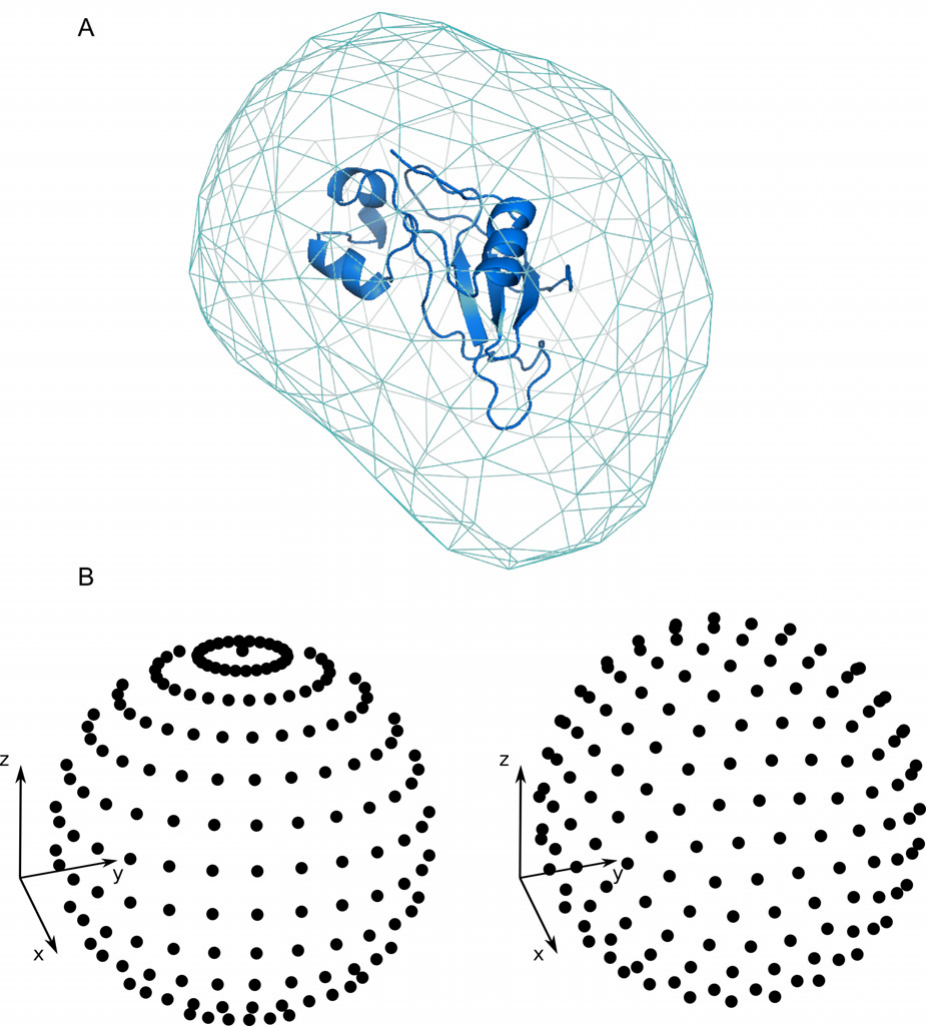
**Initial distribution of points**. A) A triangular mesh of points on the expanded surface of the barnase protein (PDB code 1bgs). B) On the left, a naïve distribution of points over a sphere based on uniform sampling of polar coordinates. On the right, a uniform distribution based on the method used in this work [18].

In order to define the different molecule orientations (as described later), we first needed to generate a uniform distribution of *N *points around each molecule. The total number of points *N *depended on the desired sampling resolution, and was calculated as follows. A triangular mesh (with equilateral faces of size *ρ*) was generated on the expanded molecular surface (figure 1A). In this way, the number of vertices of the mesh defines the number of points *N *that we need to distribute to get a sampling resolution *ρ*. The expanded molecular surface helped to overcome details on the molecular surface that could otherwise introduce error in the mesh generation, and was calculated by using MMTK module MolecularSurface [15] with a probe of radius 14 Å. This value is roughly the radius of gyration for a medium-sized protein, so the resolution *ρ *can be visualized as the distance between the center of mass of different positions of a medium-sized protein in contact with the given molecule. Once we calculated the number of points *N *that we needed to generate in order to do the sampling at a given resolution *ρ *and according to the geometrical characteristics of the protein, the next step was to distribute the *N *points over a sphere around the molecule, so that the orientations generated from these points could be uniformly distributed in the rotational space (on the contrary, the rotations directly based on the mesh points would not be uniformly distributed).

The problem of uniformly distributing a given number of points over a sphere has been studied over centuries, and constitutes one of the mathematical challenges of the XXIst century [16]. In spite of being of critical importance in many scientific disciplines, such as physics, chemistry, and biology, no analytic solution is possible. A naïve approach, based on a uniform exploration of the spherical coordinates, generates a distribution strongly biased to the poles, as can be seen in figure 1B. However, there are geometric algorithms that produce asymptotically correct solutions (i.e. they provide uniform distributions when the number of points tends to infinite) [17]. For the sake of simplicity, we have used here previously reported tables containing the distribution of point unit charges that minimizes the potential energy over a unit sphere (tables are available for *N *< 133, and *N *= 192, 212, 272, or 282) [18]. When these tables were not available, we used an algorithm based on geometrical considerations [19]. The method supposes that when the number of points is high, there is one set of points uniformly distributed that defines a tiling of the sphere by identical squares. Considering that each square edge has a certain Euclidean length, the method is able to distribute almost uniformly any number of points. This can be seen in figure 1B (on the right).

We used these uniform distributions of points around receptor (*P*_*rec*_) and ligand (*P*_*lig*_) to generate uniformly sets of rigid-body docking orientations by using three different methods, as explained below.

#### Rotational sampling method RRT ()

**Figure 2:**
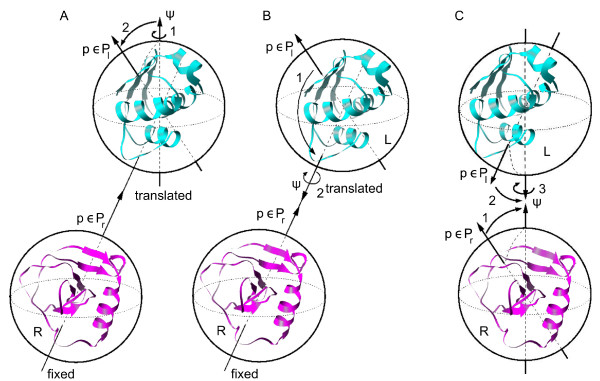
**Orientational sampling strategies used in this work**. (A) *RRT *method; (B) *TRR *method; (C) *RRR *method. See text for details.

The first method we devised to generate uniform orientations for receptor and ligand molecules is described as follows. We first generated a series of rotations for the ligand from a distribution of points over a sphere *P*_*lig *_using a method based on Arvo's work [20]. Each ligand rotation *R*_*lig *_was generated by *i*) first performing a rotation of the molecule by a specific angle *ψ *around the  axis that went through the center of mass of the molecule, and *ii*) then rotating the molecule so that the north pole pointed towards a point *p *∈ *P*_*lig *_(figure 2A). In order to ensure a uniform distribution of such defined rotations, the distributions of the angle *ψ *∈ [0, 2*π*] and the points *p *∈ *P*_*lig *_must be uniform, and the number of sampled angle values must correspond to the square root of the number of points in *P*_*lig*_. We have generated uniform orientations for receptor and ligand molecules as follows. Once the rotations for the ligand *R*_*lig *_were generated as above described, then, for each of them the ligand was translated around the receptor using a uniform set of spherical coordinates (*r*, *ϕ*, *θ*) computed from the uniform set of points *P*_*rec *_around the receptor (figure 2A). Thus, the direction of the translation was defined by the pair of angles *ϕ*, *θ *(i.e. the center of mass of the ligand was placed in the axis defined by each point in *P*_*rec *_and the center of mass of the receptor), and then the value of the radial coordinate *r *that brings both molecules in contact was computed by a new method described later (see section "Optimal distance between interacting molecules").

#### Rotational sampling method ()

In this second method, the ligand was first translated using the pairs of polar angles *ϕ*, *θ *defined from the uniform set of points *P*_*rec *_around the receptor (i.e. the ligand was translated so that its center of mass was placed in every axis defined by the receptor center of mass and each receptor point *p *∈ *P*_*rec*_). For each ligand position, the ligand was rotated around its center of mass so that every ligand point *p *∈ *P*_*lig *_ended in the axis that joined the centers of mass of the two molecules (figure 2B). Finally, the ligand was rotated around the axis that joined the centers of mass of both molecules. In order to ensure a uniform sampling, as in the first method, the distribution of the angle *ψ *∈ [0, 2*π*] and the number of sampled angle values must correspond to the square root of the number of points in *P*_*lig *_(in a previously described method based on ICM software, http://www.molsoft.com, surface points around receptor and ligand were used in a similar manner to define the translation and orientation of the ligand, but a fixed angle of 60° was used there for the final set of rotations [21]). Finally, for each orientation, the value of the radial coordinate *r *that brings both molecules in contact was computed as described later (see section "Optimal distance between interacting molecules").

#### Rotational sampling method RRR ()

In this last method, both receptor and ligand were rotated so that each one of their surface points *p*_*rec *_∈ *P*_*rec *_and *P*_*lig *_∈ *P*_*lig *_were placed in the axis that joined their centers of mass ( axis), being thus each molecule facing towards the other one. Then, the ligand was rotated around the mentioned axis (figure 2C). In order to ensure a uniform sampling, as in the other two methods, the distribution of angle *ψ *∈ [0, 2*π*] must be uniform, and the number of sampled angle values *ψ *must correspond to the square root of the number of visited ligand points (those in *P*_*lig*_). Finally, the distance *r *between the centers of mass of the two molecules was computed as described in section "Optimal distance between interacting molecules". This is in practice very similar to method *TRR*, but it reproduces in a more "natural" way the actual rotational movement of both molecules when they are interacting.

We have to note that there are other reported methods to sample rigid-body docking orientations [8,22]. For instance, Mitchell's method [22] is implemented in well-known FFT-based docking programs like ZDOCK [6]. However, these programs usually keep a fix number of rotations for all proteins, which implies that large interfaces are sampled with lower efficiency in Euclidean distance. With our approach (i.e. defining uniform points over a sphere and then using them to generate rotations) we wanted to explore the possibility of uniform rotational sampling with fixed Euclidean resolution at the sampled interfaces independently on the size of the molecules. Our RRR rotational sampling is very similar to the method of ref [8], but they do not include the mutual receptor-ligand twist angle (*ψ*) in the formalism for uniform sampling. For instance, in their manuscript they perform a test using 492 vertices and 72 twist increments of angle *ψ *(about 5° angular resolution). However, for a uniform rotation plan given 492 vertices (around 10° angular resolution) only  twist increments would be required.

### Optimal distance between interacting molecules

**Figure 3:**
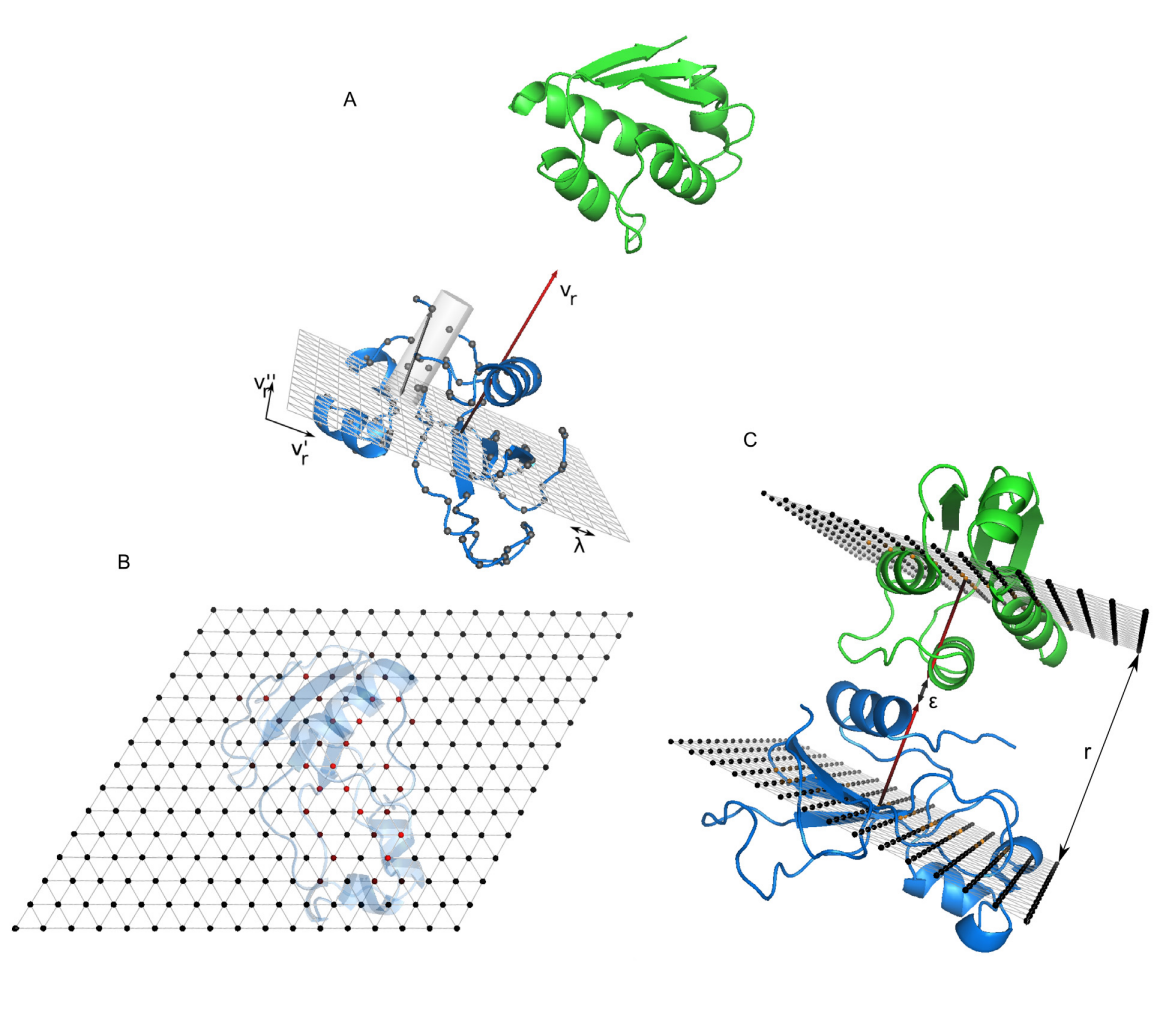
**New method for determining the optimal separation between the interacting molecules, for each sampled orientation**. (A) A triangular grid (orthogonal to the direction of the interaction *v*_*r*_) is used to generate a 2 D projection of the receptor (in blue ribbon) surface, by computing the distance from each grid point to the furthest surface backbone atoms (grey spheres). B) The grid points are shown here coloured according to the distance to the surface backbone atoms from black to red (red indicates maximum distance to the surface). C) This process is repeated for the ligand molecule, and the two grids are summed up. The value of the maximum element of the sum matrix (plus some fixed extra space ϵ to account for the volume of the side chains) will define the final separation distance *r *between the centers of mass of the molecules.

For each receptor/ligand orientation, we had to compute the optimal distance *r *between the centers of mass of the molecules. For this, we projected the molecular surfaces of the molecules as 2 D grids on the planes defined through the geometric center of receptor and ligand, respectively, both perpendicular to the axis that joined the geometric centers of the two molecules. The distance from each grid point to the molecular surface was calculated, thus generating a distance-to-surface matrix that represents the grid-projected surface for each molecule. Then, the resulting distance *r *was obtained by the maximum element of the sum of the distance to-surface matrices of receptor and ligand (figure 3). In order to speed up calculations, we computed these simplified grid-projected surfaces without transforming the molecular coordinates. This made the practical implementation for the last rotational sampling method slightly different from that of the other two methods. The details are explained below.

For the first two rotational sampling methods, *RRT *and *TRR*, rotations and translations were applied only to the ligand, so the receptor was kept fixed. We first generated a grid representation of the molecular surface for the receptor, as follows. Let  be the direction that points from the center of mass of the receptor to a given point *p *∈ *P*_*rec *_(i.e. it is the direction that joins the centers of mass of both molecules when ligand is translated to that given point *p *as described before). We defined  and  to form a basis of the perpendicular plane to , so that we generated an equilateral triangular lattice of step *λ *with them (fig. 3A). The equilateral triangular lattice provided a closer packing than a square one, and hence a better resolution. We used a lattice of size 17 × 15 cells, with *λ *= 3.5 Å. Then, for each grid point we took the farthest surface backbone atom (defined as those with a non-zero accessible surface area) inside the cylinder of radius *λ*/2, centered at that point with axis parallel to  (fig. 3A) (the mathematical condition is that the cross product between  and the vector that points from the grid point to the atom has a value smaller than ). The distance between the grid point and the selected atom is calculated for each grid point, so that we obtained a distance matrix that mimicked the shape of the receptor from each grid point (fig. 3B).

Applying the algorithm to the ligand is analogous. Given the rotation matrix *R*_*lig*_, the ligand has its interaction on the direction given by:(1)

To find a basis  and  of its perpendicular plane, we used the transformation in equation 1 over  and . Then we generated a grid defined through the center of masses of the ligand and the corresponding distance matrix for the ligand (as explained before for the receptor).

Finally we summed both matrices, selected the maximum element that had both summands no null and added ϵ = 4.7 Å (arbitrary value selected from previous tests; data not shown) to this quantity in order to account for the extra volume required to include the side-chains of both proteins. The resulting value was assigned as to *r*, accounting for the distance between centers of mass that kept the surfaces in contact (figure 3C).

For the third rotational sampling method, *RRR*, both receptor and ligand molecules were rotated so that the interaction was always on the  axis. Then, in order to generate the grid-projected surface of the receptor we took the direction *v*_*rec *_(the direction from the geometric center of the receptor to the given point *p *∈ *P*_*rec*_), and the difference with the other two methods for generating rotations is that now we need to generate the vector basis depending on *R*_*rec *_as:(2)

Similarly, for the ligand, we had  as the direction corresponding to the given point *p *∈ *P*_*lig*_, and the vector basis:(3)

The rest was computed as above described.

### Scoring the conformations

We have used pyDock [13] for the final scoring of the orientations generated by RotBUS. The function, based on electrostatics and desolvation, with weighted van der Waals term, is specially suited to study rigid-body protein-protein docking. In the original pyDock benchmark [13], a weight of 0.1 for van der Waals term was optimal to tolerate the small number of atomic clashes found in the FFT-generated rigid-body docking poses. However, in RotBUS docking orientations might not have such good geometric complementarity as in FFT-based approaches. Thus, in order not to filter out acceptable orientations, we had to consider lowering van der Waals weight (see Results for further details).

Given the large amount of docking orientations generated by RotBUS, pyDock evaluation became computationally too expensive at high resolution (e.g. *ρ *= 9 Å). Thus we devised a fast residue-based desolvation scoring. Using the atomic parameters *σ*_*i *_in a previous work [21], we have first calculated the contribution of each surface residue to the desolvation energy in the individual proteins, :(4)

Then, for each docking orientation the total desolvation energy was computed by summing up the residue contributions in all intermolecular contact residue pairs (i.e. residues from each molecule that have at least one atom within 8 Å distance), as in the following equation:(5)

The contribution of each residue thus depended on its intrinsic contribution (precomputed in the individual proteins) and on the number of contact pairs formed with residues from the partner molecule. The concern with this approach was that the residues involved in several pairs might be over-represented and thus could contribute excessively to the desolvation energy. However, we checked that in this method (as opposed to that of considering each interface residue only once) the resulting energy correlated better with the originally described atom-based desolvation energy. The main advantage is that accessible surface area was calculated only once (on the individual proteins), and thus during the scoring process only distances needed to be computed. This scoring method showed to be much faster than pyDock and therefore was used as a pre-pyDock scoring filter.

### Data set for benchmarking

We analyzed the predictive capabilities of RotBUS, using a standard benchmark for docking formed by 84 protein-protein cases with known X-ray structures both for the unbound and the bound subunits [12]. In order to perform a test as much realistic as possible, the orientation of the initial structures was randomized (the structures in the benchmark are provided as superimposed to the x-ray structure of the complexes, which could yield artificial better results). The success rates of the predictions were defined by the percentage of test cases in which at least one acceptable docking solution was found within the top *N *solutions. A nearnative or acceptable docking solution was defined as the one that had below 10 Å of RMSD of the ligand C-alpha atoms with respect to the corresponding ones in the reference structure, after superimposing the receptor molecules. In order to focus our test onto the rigid-body results, for the first test regarding the number of near-native solutions at different sampling conditions, the complex reference was formed by the unbound subunits superimposed onto the complex structure (thus avoiding the high RMSD values that cases with large unbound-bound differences could have even with correct docking orientations).

## Results and Discussion

### Sampling efficiency by the different rotation methods

We have devised three different methods for rigid-body sampling (see Methods), based on uniform rotations, and have evaluated the quality of the generated docking orientations on a widely-used benchmark set formed by 84 protein-protein complexes [12].

First we analyzed the percentage of cases with near-native docking solutions (defined with respect to a reference structure formed by superimposing the unbound subunits onto the complex structure) generated by each method, at different levels of resolution (see table 1). The resolution value *ρ *indicated the minimal distance between the grid points on the expanded surface that were used to define the number of rotations, as described in Methods. The resolution value can be intuitively related to the distance between the geometrical centers of the different ligand orientations sampled during the procedure. For instance, a 14 Å resolution means that, for standard-sized molecules, the docking orientations would be uniformly sampled so that the minimal distance between the geometrical centers of the ligands (after superimposing the receptors) would be 14 Å, while the contacting surfaces would be approximately 9 Å separated (on the other side, at 9 Å resolution, the contacting surface would be approximately 6 Å separated). In order to compare with standard angular resolution units, a resolution of 14 Å would be equivalent to 14.0° for a big protein (1de4 ligand, 1278 residues) and to 28.4° for a small one (1eaw ligand, 58 residues). On the other side, a resolution of 9 Å is equivalent to 8.5° for a large protein (1de4 ligand, 1278 residues) and 17.5° for a small one (1eaw ligand, 58 residues). In this way, for the largest cases, at 9 Å resolution we have better surface sampling than with the standard 12° resolution used in FFT-based approaches.

**Table 1:** Docking results with different rotational sampling methods.

*ρ *(Å)	% of cases with solution			# conformations
	***RRT***	***TRR***	***RRR***	
		
9.0	100.0	100.0	100.0	1196123
12.5	98.8	100.0	100.0	187110
13.0	95.2	94.0	97.6	152156
13.5	92.8	90.5	95.2	123687
14.0	92.8	88.1	91.7	100902
14.5	88.1	88.1	90.5	82903
15.0	84.5	84.5	84.5	69395
15.5	81.0	78.6	81.0	57643

Resolution (*ρ*), percentage of cases with near-native solutions generated by each method and average number of generated conformations.

The three methods had similar results. In all of them, the number of cases with near-native docking solutions increased at higher resolution values (i.e. those with smaller grid cell size), but obviously the number of total docking poses also increased. Given that the *RRR *method gave slightly best results for high +resolution (below 14 Å), and since it was more intuitive (as it directly described the docking orientations based solely on molecular rotations without needing any translation), we decided to use it in this work.

**Figure 4:**
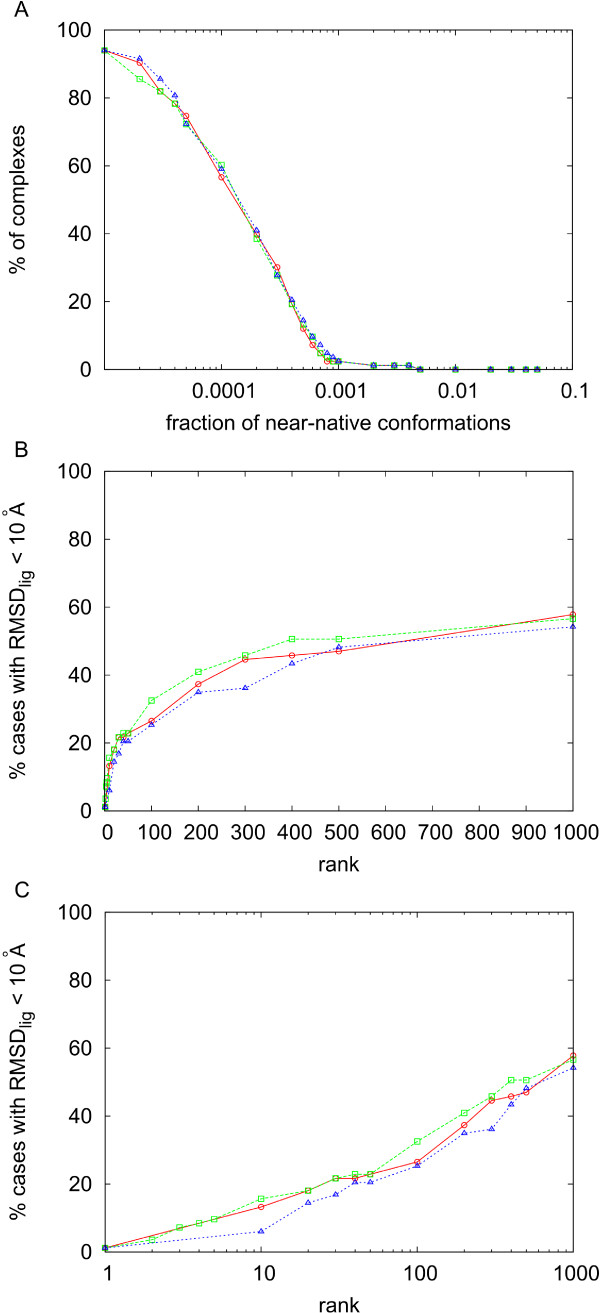
**Analysis of the RotBUS orientational sampling**. A) Percentage of cases with concentration of near-native solutions (9 Å ligand RMSD with respect to a reference formed by the unbound molecules superimposed on the complex structure) above the value indicated in abscissas for the rigid-body docking sets generated by *RRR *method at 9 Å (blue), 12.5 Å (green) and 14 Å resolution (red). B) Success rates (i.e. percentage of cases with near-native solutions -10 Å ligand RMSD with respect to the complex structure- within the top ranked docking poses indicated in abscissas) after scoring by pyDock the docking sets generated at 14 Å resolution, using weighting factors for the van der Waals term 0 (red), 0.02 (green), and 0.1 (blue). C) Same as in B but in logarithmic scale.

Because of the systematic sampling, the number of near-native solutions was proportional to the total number of docking poses, and this in turns depended on the resolution. At 9 Å, all cases had near-native conformations, although the total number of generated docking poses was quite high in average (circa 1.2 million). On the other side, at 12 Å resolution, the average total number of docking poses was more reasonable (below 100,000), while the percentage of cases with near-native solutions was still high (77 out of 84 cases). Moreover, we can see in figure 4A that the concentration of near-native solutions at 14 Å at 12.5 Å and at 9 Å resolution are very similar. This is important, as we have recently reported that the concentration of near-native solutions is a key factor for pyDock scoring success [23].

For the only purposes of exploring the number of near-native solutions found at the different sampling conditions, as described in this sub-section, the ligand RMSD of each docking pose was calculated with respect to a reference formed by the unbound structures superimposed onto the complex structure (although we have to note that for all the success rate results given later, we have calculated the ligand RMSD with respect to the X-ray structure of the complex, as standard). In this way, conformational changes in the subunits were ignored so that we could focus on the capability of the sampling algorithm to find correct orientations. There are 77 cases that had near-native solutions at 14 Å resolution, but we needed to increase resolution up to 12.5 Å in order to have near-native solutions for all the remaining 7 cases. When RMSD was computed with respect the complex structure, the only case in the combined set (14 Å/12.5 Å) that did not have any near-native solution (i.e. RMSD < 10 Å) was 1h1v (one of the set of cases run at 12.5 Å resolution), in which the unbound ligand was 14 Å from the bound one. For these 83 complexes with near-native solution, there was an average of 15.3 near-native conformations (with a standard deviation of 13.2). Moreover, 53 of these cases had 10 or more acceptable solutions (defined with respect to the complex structure). For comparison, FTDock (12° angular resolution, 0.7 Å grid size, with electrostatics, generating 10,000 conformations per complex) failed to find a near-native solution in 17 cases, found only one nearnative solution in 11 other cases, and found 10 or more acceptable solutions in only 15 cases. All these data indicate that the method presented here can properly sample the 2-rigid body conformational space, with optimal parameters for the number of rotations, radial distance *r *and resolution.

### Scoring with pyDock

Having proven the efficiency of the new sampling method in generating rigid-body docking orientations, we next focused on the scoring, with the goal of accurately identifying the near-native docking solutions (based on ligand RMSD computed with respect to the complex structures, as standard). Figures 4B,C show the performance of pyDock scoring on the solutions generated by the *RRR *method at 14 Å resolution (except for the 7 cases with no near-native solution, in which we used 12.5 Å resolution). We checked the use of van der Waals and there was a little improvement with 0.02 weight. As can be seen in figure 4B,C a weight of 0.02 gave the best success rate for top 10 solutions, and it gave better results for all rank values. This small van der Waals contribution was sufficient to remove false positives in which electrostatics was artificially high. Moreover, imposing a van der Waals cutoff to avoid cases with high van der Waals values did not change the results (data not shown). On the other side, higher van der Waals weights were not helping either, probably because of the noise derived from the rigid-body assumption (as discussed in previous work [13]). The main problem is that at 14 Å resolution, the number of docking poses was too high for the practical application of pyDock (the average computational time was more than 130 hours per case). For this reason, we further explored the use of a fast residue-based desolvation (see Methods) as a first filtering step in order to reduce the number of docking poses to be scored by pyDock.

### Filter with fast solvation energy

**Figure 5:**
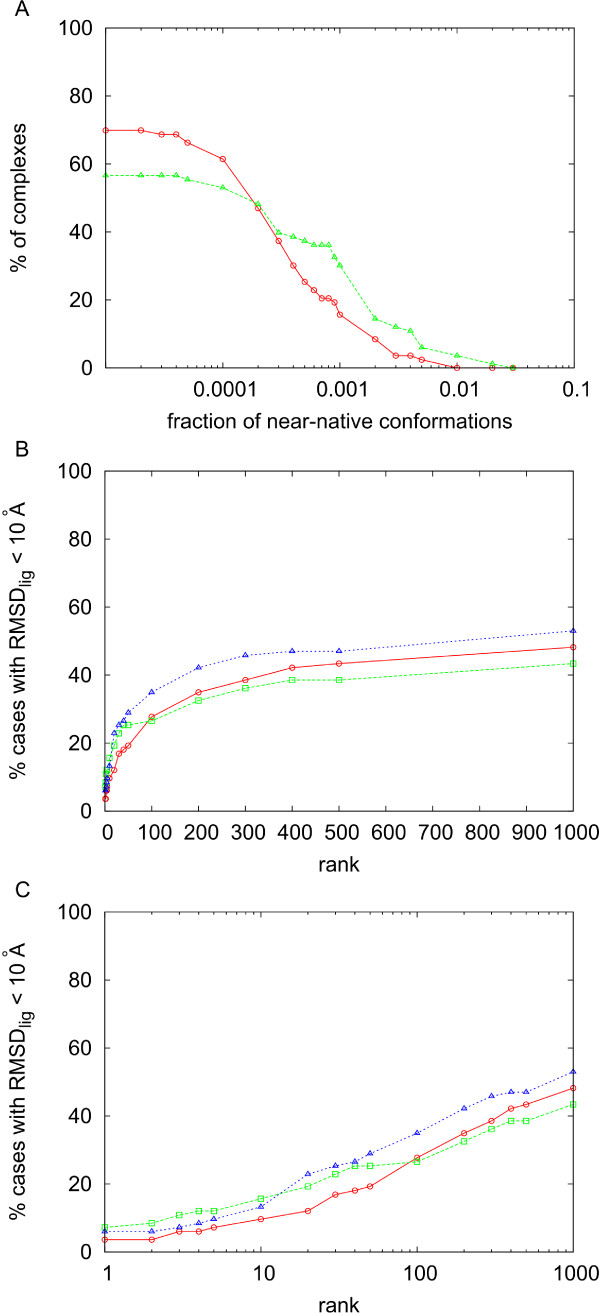
**Analysis of the residue-based desolvation filtered subsets**. A) Percentage of cases with concentration of near-native solutions (10 Å ligand RMSD with respect to a reference formed by the unbound molecules superimposed on the complex structure) above the value indicated in abscissas at 9 Å (green) and 12.5 Å/14 Å resolution (red), when only the top 1% and 10% docking poses with the best residue-based desolvation values are considered. B) Success rates (near-native solutions defined with respect to the complex structure) after scoring by pyDock (*w*_*vdw *_= 0.02) the top 1% docking poses with the best residue-based desolvation of the docking sets generated at 9 Å and 12.5 Å/14 Å resolution (in red and green, respectively), and the top 10% docking poses with the best residue-based desolvation of the docking sets generated at 9 Å resolution (blue). C) Same as in B but in logarithmic scale.

First we studied the most efficient filtering protocol with the new residue-based desolvation in terms of recovery of near-native solutions. When we applied the fast desolvation to the sets derived at 9 Å and 12.5 Å/14 Å resolution, we could keep a similar number of total docking poses by selecting the top 1% in the 9 Å resolution set (yielding an average of about 11800 docking poses) or the top 10% in the 12.5 Å/14 Å resolution set (yielding an average of about 11300 docking poses). However, although the final number of selected conformations was similar, we saw an important difference: the concentration of near-native solutions (defined with respect to the unbound subunits superimposed on the complex structure) was higher after selecting the top 1% lowest-desolvation orientations in the 9 Å resolution set (figure 5A). In these conditions, the number of near-native solutions found in the top 2000 conformations generated by RotBUS is in many cases significantly higher than when generated by FTDock (see [Additional file 1]).

In figure 5B,C we can see that filtering the set at 12.5 Å/14 Å resolution with the new residue-based desolvation permits a fast removal of 90% of the docking poses, without losing too much efficiency in the pyDock scoring. Computational times for a small case like PDB 1eaw were less than 2 minutes for the orientation search and 40 seconds for the residue-based solvation calculation on a 2.4 GHz Dual Core AMD Opteron CPU. For one of the largest cases (PDB code 1de4) these times increased up to 32 and 52 minutes, respectively. In any case, given that most of the time is spent in pyDock scoring, removal of 90% of the poses makes the total procedure around ten times faster.

Besides, given that selecting the top 1% at 9 Å resolution seemed more efficient in terms of recovery of near-native solutions, we have analyzed pyDock success rates using this strategy (from now on, near-native solutions are defined based on ligand RMSD computed with respect to the complex structures, as standard). In figure 5B,C (and in [Additional file 2]) we can see the results for 9 Å resolution after selecting the 1% docking poses with the lowest residue-based desolvation plus final scoring by pyDock (with van der Waals 0.02). For low ranks, 9 Å resolution (1% filtering) was clearly better than 14 Å resolution (10% filtering).

When selecting instead the top 10% (9 Å resolution), the results were slightly better at high rank values, but they were worse at low rank values (figure 5). At 9 Å resolution, computational times for a small case like PDB 1eaw were 10 minutes for the orientation search and 3 minutes for the residue-based solvation calculation on a 2.4 GHz Dual Core AMD Opteron CPU. For the exceptionally large case PDB 1de4 these times dramatically increased up to 5 and 10 hours, respectively. In any case, these times were smaller than those of FTDock (for 1eaw and 1de4, 6 and 60 hours respectively) and pyDock (the latter depending on the number of solutions to score). Considering that pyDock scoring is 10 times faster with 1% filtering than with 10% filtering, this is clearly the strategy of choice.

### Integration of RotBUS and FTDock sets

**Figure 6:**
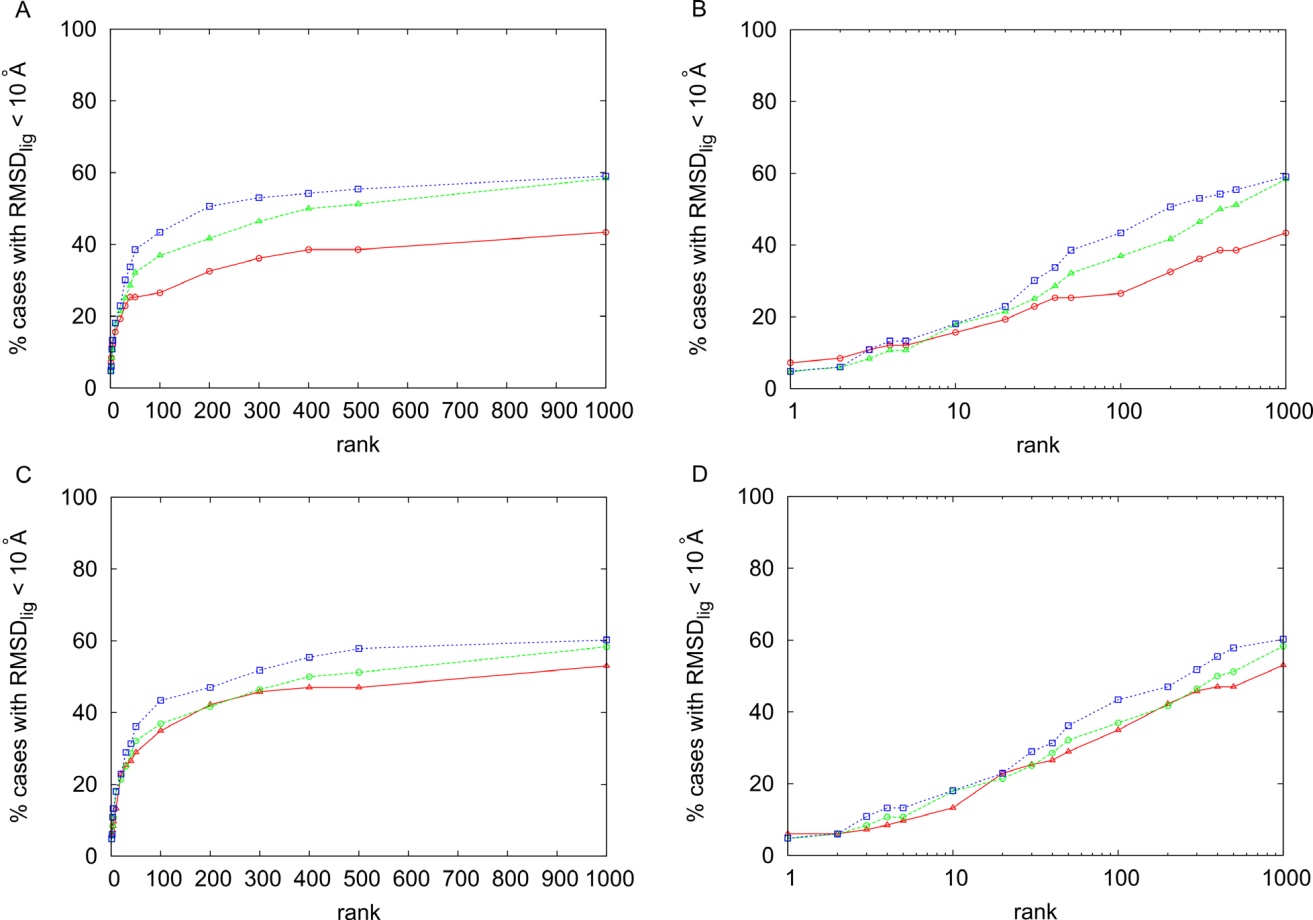
**Analysis of the combined RotBUS and FTDock sets**. Success rates for RotBUS (generated at 9 Å resolution and scored by pyDock with 0.02 van der Waals weight; in red), FTDock (scored by pyDock with 0.1 van der Waals weight; in green), and the combined sets from RotBUS and FTDock, in which the scoring values of the RotBUS docking poses have been weighted by a factor of 0.5 (in blue). A) For RotBUS, only the top 1% docking poses with the best residue-based desolvation values were considered. B) As in A but in logarithmic scale. C) For RotBUS, only the top 10% docking poses with the best residue-based desolvation values were considered. D) As in C but in logarithmic scale.

In figure 6 and [Additional file 1] we have compared the results of RotBUS plus pyDock with those obtained from FTDock plus pyDock. For rank values of 10 or below, the results are very similar. However, for larger rank values the success rates are better in FTDock. We have explored whether both methods could be complementary and thus generate good solutions in cases in which the other one could have difficulties, and vice versa. We have checked that in many cases the number of near-native solutions in the top 2000 conformations is high when generated by RotBUS or by FTDock, but not by both simultaneously (see [Additional file 1]). Figure 6A,B shows the success rates of the combined docking sets from i) 9 Å resolution plus 1% filtering, scored by pyDock with 0.02 van der Waals, with the final value weighted by a factor of 0.5, and ii) FTDock, scored by pyDock with 0.1 van der Waals. Other weighting factors for the pyDock scores of the RotBUS poses did not improve the results (see [Additional file 3]). With 9 Å filtered at 10%, the results did not improve (figure 6C,D). We checked that considering a fix number of top solutions (e.g. 10,000) after solvation filtering gave similar results (data not shown).

RotBUS with residue-based desolvation filtering in combination with FTDock gave better general results than each method on their own, especially for rank values over 50. However, we saw specific examples that were very dependent on the choice of the method. For instance, there were cases that had poor results with RotBUS (9 Å resolution) filtering at 1% residue-based desolvation, but had good results with FTDock, such as those with PDB code 1ay7, 1b6c, 1buh, 1ml0, 2btf, and 2jel. These cases can improve the results if we apply 10% filtering, which indicates that for them, geometrical complementarity rather than desolvation is the important factor to detect the near-native solutions. On the contrary, there were cases that had good results with RotBUS at 1% filtering, but not with FTDock, such as those with PDB code 1de4, 1n2c, 2hmi, and 2qfw. Moreover, the results did not further improve with 10% filtering. In these cases, desolvation seems to be determinant (indeed, in all of these cases, except the ligand of 1n2c, the interacting molecules had highly significant ODA values [24]), and thus our residue-based desolvation is helping to identify the near native solutions even if they do not have optimal geometrical complementarity or are too large for FTDock sampling. For these cases, the FTDock results could improve by inclusion of some solvation descriptor during the FFT-based search. Indeed, we have checked that the program ZDOCK 3.0 [25], which includes solvation, gives for these cases much better results than FTDock (rank 21, 72, 544, 1 for 1de4, 1n2c, 2hmi and 2qfw, respectively), which suggests that FFT-based approaches can cover the same sets of orientations of RotBUS with appropriate inclusion of solvation. In general, in order for RotBUS sampling to have the same success rates as FTDock (especially at high rank values), we would need to use 9 Å resolution at 10% filtering. However, it seems much more efficient to combine RotBUS at 1% filtering with FTDock if we need to produce more near-native solutions in a selection of docking poses for further refinement, for instance.

### Success rates by size...does it improve FTDock?

We have studied the success rates of FTDock and RotBUS according to the size of the proteins. To be consistent with previous studies we have used the size of the grid, *s*, generated by FTDock [23], according to:(6)

where  is the maximum radius of the receptor (distance from the center of coordinates of the receptor protein to its farthest atom),  maximum radius of the ligand, and *δ *the resolution of the grid used by FTDock (0.7 Å in our case). As we recently saw, pyDock had very good results on the docking poses generated by FTDock for small cases (*s *< 150), but very bad results for the large cases (*s *> 250). The reason was that for these cases, FTDock had problems in generating a sufficient number of acceptable docking solutions. We have found here that FTDock is giving slightly better pyDock success rates than RotBUS for small cases (*s *< 200). But for large cases (*s *> 250), top 100 success rates were by far much better when docking poses were generated by RotBUS (33.3%) than by FTDock (0%). Some interesting examples are the cases with PDB code 1de4, 1n2c and 2hmi, which had pyDock ranks between 12 and 32 when docking poses were generated by RotBUS, although they did not have any acceptable solution generated by FTDOCK. It is clear that the limitations of FTDock with respect to the large-sized cases can be overcome with RotBUS efficient sampling.

### RotBUS and beyond

When external information about the protein-protein interaction is provided, e.g., computationally predicted hot spots, residue conservation or experimental data, the search can be restricted to defined sub-zones of the subunits. With RotBUS, this can be easily done by checking whether the vectors point to the expected interacting surfaces for receptor and/or ligand, respectively. If they do not, the distance *r *is not calculated and the orientation is disregarded. This dramatically reduces computational costs, making possible the study of larger cases.

**Figure 7:**
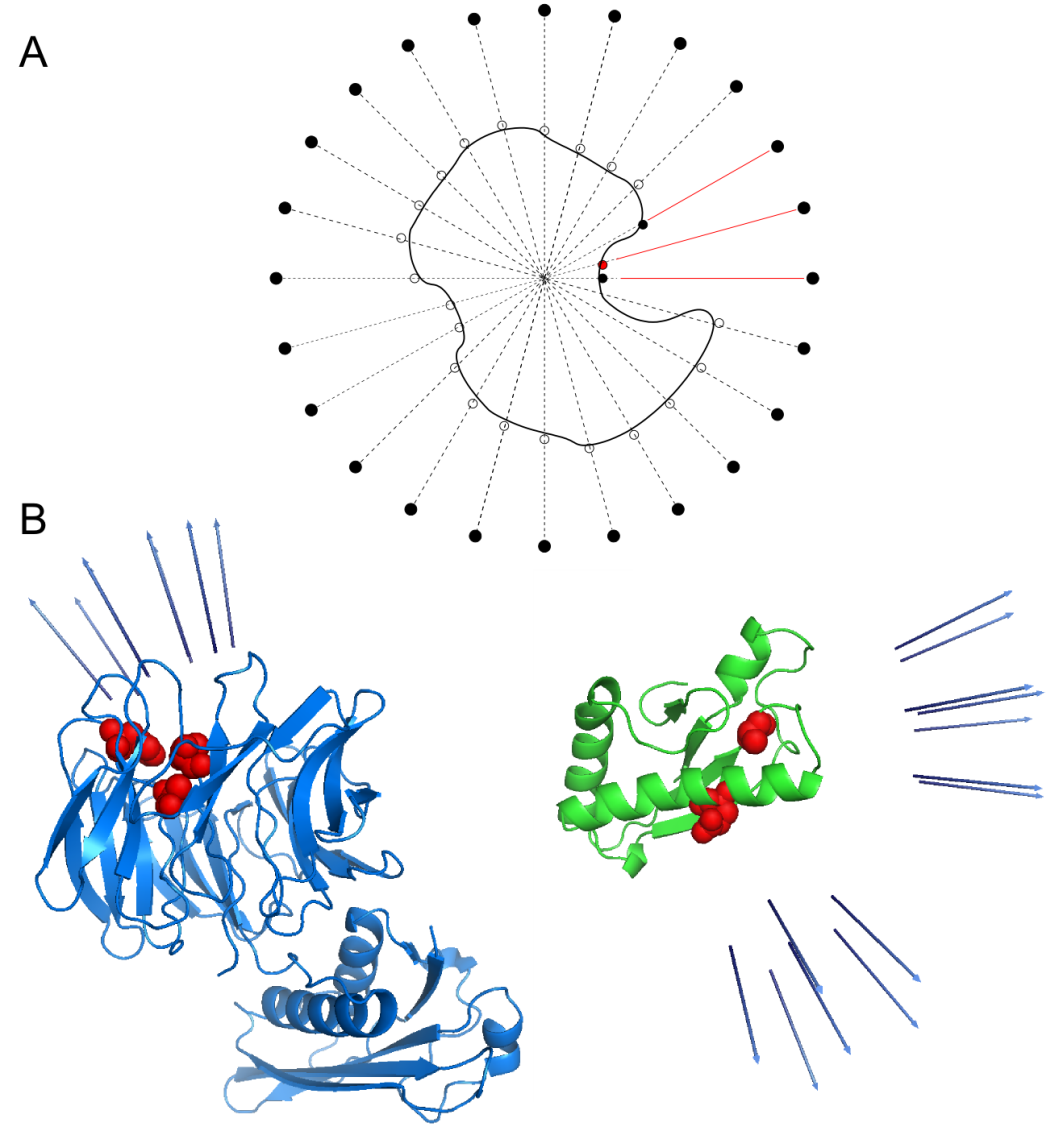
**Restricted sampling with RotBUS**. A) Diagram of the chosen points to be explored, when there is available external information on the expected binding regions. Red dot is the expected interface residue. Red lines represent the directions selected for sampling (the one corresponding to the expected interface residue, and the neighbour directions). B) CAPRI target 26 receptor and ligand respectively. The residues depicted in red are expected to be in the interface, the arrows in blue account for the selected directions to be explored in both receptor and ligand molecules.

**Figure 8:**
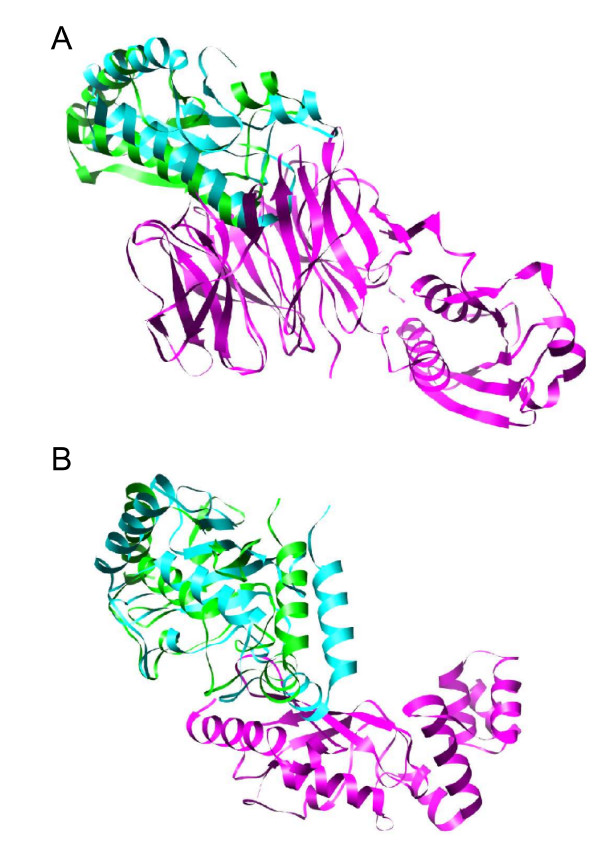
**Results of restraint-filtered RotBUS for the CAPRI targets T26 and T27**. A) Near-native solution (rank 1) for the complex T26, with RMSD_*lig *_= 6.9 Å. Receptor in magenta, ligand in cyan and reference ligand in green. B) Near-native solution (rank 25) for the complex T27, with RMSD_*lig *_= 7.6 Å. Same colour code as in A.

As an example of practical application, we have performed this restricted search on two CAPRI http://www.ebi.ac.uk/msd-srv/capri[26] targets, T26 (TolB/Pal) and T27 (Ubc9/E2-25K). We first projected the spherical uniform set of points (which represents all the directions to be explored a priori) on the surface of each subunit. For each residue expected to be at the interface, we have selected the direction corresponding to the closest projected point (figure 7A). We selected also the nearest neighbours of the corresponding direction in order to ensure a wider sampling. The expected interface residues (easily obtained from previous literature knowledge) for CAPRI target T26 receptor TolB were His246, Ala249 and Thr292, while for ligand Pal were Ala88 and Glu102 [27]. For target T27, the expected interface residues in receptor Ubc9 were Gln126, Gln130, Ala131, Glu132, Tyr134, and T135 [28], while the ones for ligand E2-25K were Arg8, Lys10, Arg11, Phe13 and Lys14 [29]. For target T26, the residue restraints reduced the number of explored docking poses from about 780,000 to only 1,300 orientations, at a sampling resolution of 9 Å (no filtering with residue-based desolvation). For target T27, the original sampling of about 820,000 orientations was reduced to only 6,000 ones with the restricted search. When pyDock was applied to these restraint-filtered sets of docking poses, a near-native solution was detected at rank 1 for T26, as can be seen in figure 8A (for comparison, in our CAPRI submission, unrestricted docking obtained a near-native solution at rank 19, which was improved to rank 6 with pyDockRST). For T27, a near-native solution was found at rank 25 using the same restricted-search method (for comparison, in our CAPRI submission, unrestricted docking obtained a near-native solution at rank 977, which was improved to rank 5 with pyDockRST) [30].

Similarly, our sampling method could be easily extended or adapted to other algorithms. For instance, flexibility can be implemented by loading several structures of the proteins when the radial coordinate is being computed. These structures could be generated, for example, from a molecular dynamics ensemble. In this way, future implementations of this method may provide an efficient approach to the problem of flexibility in protein-protein docking, especially for cases in which large conformational changes invalidate other more standard algorithms such as FFT-based methods. Moreover, this method could be implemented in combination with any other conformational search protocol, for example as part of a Monte Carlo strategy to perform simple minimizations or thermodynamic calculations.

## Conclusions

We have presented here a new systematic approach to generate rigid-body orientations of a receptor-ligand system, based on three novel algorithms: i) uniform definition of rotations of receptor and ligand; ii) fast computing of optimal distance between proteins; and iii) fast filtering with residue-based desolvation. The method generates docking orientations at low computational cost and good efficiency, and can be complemented with those generated by other methods (e.g. FFT-based). The final scoring with the previously developed pyDock function yields competitive success rates and opens the door to efficient treatment of flexibility by using pre-sampled ensembles or on-the-fly conformational search methods.

## Authors' contributions

JFR devised the concept, directed the research and finalized the draft. AS performed the calculations and drafted the paper. All authors read and approved the final manuscript.

## Supplementary Material

Additional file 1 **Number of near-native structures in the top 2000 conformations generated by RotBUS and FTDock**. We show the number of near-native structures in the top 2000 conformations generated by the method presented here (RotBUS 9A resolution, 1% lowest residue-based desolvation), as compared to when generated by the well-known FFT-based method FTDock. For the sake of completeness, we have also shown in brackets the number of near-native structures in the top 2000 conformations generated by each method after scoring by pyDock. Click here for file

Additional file 2 **Benchmark results for the RotBUS+PyDock protocol**. Docking results after scoring with PyDock the set of rigid body poses generated by RotBUS at 9 Å resolution and filtered up to 1% with best residue-based solvation. RMSD is calculated for ligand C-alpha atoms with respect to the complex structure. Click here for file

Additional file 3 **Extra weight values for the analysis of the combined RotBUS and FTDock sets**. Success rates for RotBUS (generated at 9 Å resolution and scored by pyDock with 0.02 van der Waals weight; in blue diamonds), FTDock (scored by pyDock with 0.1 van der Waals weight; in green triangles), and the combined sets from RotBUS and FTDock, in which the scoring values of the RotBUS docking poses have been weighted by different factors from 0.1 to 1. A) For RotBUS, only the top 1% docking poses with the best residue-based desolvation values were considered. B) As in A but in logarithmic scale. Click here for file
