# Supplementary material for: Protein docking by Rotation-Based Uniform Sampling (RotBUS) with fast computing of intermolecular contact distance and residue desolvation
Source: BMC Bioinformatics. 2010 Jun 28;11:352. doi: 10.1186/1471-2105-11-352 (PMC2911459; doi:10.1186/1471-2105-11-352)
Supplement: Additional file 1 — Number of near-native structures in the top 2000 conformations generated by RotBUS and FTDock. We show the number of near-native structures in the top 2000 conformations generated by the method presented here (RotBUS 9A resolution, 1% lowest residue-based desolvation), as compared to when generated by the well-known FFT-based method FTDock. For the sake of completeness, we have also shown in brackets the number of near-native structures in the top 2000 conformations generated by each method after scoring by pyDock. [file 1471-2105-11-352-S1.PDF]

Additional file 1: Number of near-native structures in the top 2000 conformations generated by RotBUS and FTDock

We show the number of near-native structures in the top 2000 conformations generated by the method presented here (RotBUS 9A resolution, 1% lowest residue-based desolvation), as compared to when generated by the well-known FFT-based method FTDock. For the sake of completeness, we have also shown in brackets the number of near-native structures in the top 2000 conformations generated by each method after scoring by pyDock.

Complex	RotBUS	FTDock
1a2k	0 (0)	0 (2)
1acb	4 (6)	1 (1)
1ahw	0 (0)	3 (0)
1ak4	13 (12)	0 (0)
1akj	0 (0)	1 (1)
1atn	2 (7)	0 (0)
1avx	0 (0)	2 (2)
1ay7	0 (0)	4 (9)
1b6c	0 (0)	2 (7)
1bgx	0 (0)	0 (0)
1bj1	4 (2)	0 (0)
1buh	0 (0)	4 (11)
1bvk	0 (1)	3 (15)
1bvn	12 (21)	8 (15)
1cgi	56 (59)	7 (20)
1d6r	0 (0)	2 (0)
1de4	0 (2)	0 (0)
1dfj	0 (0)	1 (1)
1dqj	0 (0)	1 (5)
1e6e	1 (8)	1 (4)
1e6j	2 (7)	2 (11)
1e96	1 (1)	0 (11)
1eaw	0 (0)	2 (3)
1eer	0 (0)	0 (1)
1ewy	0 (3)	13 (34)
1ezu	0 (0)	0 (1)
1f34	0 (0)	1 (2)
1f51	0 (0)	2 (6)
1fak	0 (0)	0 (0)
1fc2	4 (4)	0 (0)
1fq1	0 (0)	1 (0)
1fqj	0 (0)	0 (1)
1fsk	12 (18)	6 (8)
1gcq	0 (0)	0 (5)
1ghq	0 (0)	0 (0)
1gp2	4 (15)	0 (0)
1grn	0 (0)	2 (5)
1h1v	0 (0)	0 (0)
1he1	4 (0)	2 (0)
1he8	0 (0)	1 (0)

Complex	RotBUS	FTDock
1hia	0 (1)	4 (5)
1i2m	0 (0)	0 (0)
1i4d	17 (44)	0 (0)
1i9r	0 (0)	0 (1)
1ib1	0 (0)	0 (0)
1ibr	0 (0)	0 (0)
1ijk	0 (0)	1 (2)
1iqd	3 (20)	0 (4)
1jps	0 (0)	0 (0)
1k4c	0 (0)	0 (0)
1k5d	0 (0)	0 (1)
1kac	3 (0)	5 (0)
1kkl	9 (10)	3 (8)
1klu	0 (0)	1 (0)
1ktz	0 (0)	0 (0)
1kxp	4 (6)	1 (4)
1kxq	0 (0)	0 (1)
1m10	0 (0)	0 (3)
1mah	5 (21)	1 (6)
1ml0	0 (0)	4 (8)
1mlc	21 (35)	0 (1)
1n2c	1 (12)	0 (0)
1nca	1 (2)	0 (1)
1nsn	0 (0)	0 (2)
1ppe	27 (31)	19 (26)
1qa9	0 (0)	0 (0)
1qfw	0 (0)	0 (3)
1rlb	3 (0)	0 (0)
1sbb	0 (0)	1 (3)
1tmq	1 (10)	1 (4)
1udi	10 (22)	2 (12)
1vfb	3 (6)	0 (4)
1wej	2 (6)	0 (1)
1wq1	0 (0)	0 (0)
2btf	0 (0)	1 (3)
2hmi	0 (4)	0 (0)
2jel	0 (0)	0 (8)
2mta	0 (1)	6 (14)
2pcc	13 (33)	6 (25)
2qfw	5 (14)	0 (0)
2sic	9 (5)	2 (6)
2sni	40 (55)	7 (14)
2vis	0 (0)	0 (0)
7cei	0 (5)	6 (12)
