# Supplementary material for: Protein docking by Rotation-Based Uniform Sampling (RotBUS) with fast computing of intermolecular contact distance and residue desolvation
Source: BMC Bioinformatics. 2010 Jun 28;11:352. doi: 10.1186/1471-2105-11-352 (PMC2911459; doi:10.1186/1471-2105-11-352)
Supplement: Additional file 2 — Benchmark results for the RotBUS+PyDock protocol. Docking results after scoring with PyDock the set of rigid body poses generated by RotBUS at 9 Å resolution and filtered up to 1% with best residue-based solvation. RMSD is calculated for ligand C-alpha atoms with respect to the complex structure. [file 1471-2105-11-352-S2.PDF]

Additional file 2 - Benchmark results for the RotBUS+PyDock protocol.

Docking results after scoring with PyDock the set of rigid body poses generated by RotBUS at 9Å resolution and filtered up to 1% with best residue-based solvation. RMSD is calculated for ligand C-alpha atoms with respect to the complex structure.

Rigid-Body (63)

Complex	Cat.*	RMSD (Å)	Rank	Complex	Cat.*	RMSD (Å)	Rank
1avx	E	-	-	1ay7	E	-	-
1bvn	E	7.0	1	1cgi	E	6.0	12
1d6r	E	-	-	1dfj	E	-	-
1e6e	E	9.2	29	1eaw	E	-	-
1ewy	E	9.2	124	1ezu	E	8.0	3533
1f34	E	-	-	1hia	E	9.2	781
1mah	E	7.0	1	1ppe	E	9.1	1
1tmq	E	9.7	9	1udi	E	4.7	2
2mta	E	9.8	365	2pcc	E	8.1	1
2sic	E	8.2	205	2sni	E	6.1	1
7cei	E	9.3	258	1ahw	A	-	-
1bvk	A	6.9	289	1dqj	A	-	-
1e6j	A	7.6	140	1jps	A	-	-
1mlc	A	6.3	36	1vfb	A	6.1	21
1wej	A	9.7	3	2vis	A	-	-
1a2k	O	-	-	1ak4	O	7.9	370
1akj	O	-	-	1b6c	O	-	-
1buh	O	-	-	1e96	O	6.8	9
1f51	O	-	-	1fc2	O	8.8	784
1fqj	O	7.4	2267	1gcq	O	-	-
1ghq	O	-	-	1he1	O	5.5	3323
1i4d	O	7.1	102	1kac	O	9.1	4150
1klu	O	8.2	10495	1ktz	O	-	-
1kxp	O	9.0	1	1ml0	O	-	-
1qa9	O	-	-	1rlb	O	7.3	3220
1sbb	O	-	-	2btf	O	-	-
1bj1	AB	9.2	599	1fsk	AB	8.7	4
1i9r	AB	-	-	1iqd	AB	8.5	3
1k4c	AB	-	-	1kxq	AB	9.7	4367
1nca	AB	9.9	899	1nsn	AB	-	-
1qfw	AB	-	-	2qfw	AB	9.0	12
2jel	AB	-	-				

Medium Difficulty (13)

Complex	Cat.*	RMSD (Å)	Rank	Complex	Cat.*	RMSD (Å)	Rank
1acb	E	9.9	8	1kkl	E	8.7	91
1bgx	A	-	-	1gp2	O	5.4	110
1grn	O	-	-	1he8	O	-	-
1i2m	O	-	-	1ib1	O	-	-
1ijk	O	-	-	1k5d	O	-	-
1m10	O	-	-	1n2c	O	5.9	32
1wq1	O	-	-				

Difficult (8)

Complex	Cat.*	RMSD (Å)	Rank	Complex	Cat.*	RMSD (Å)	Rank
1atn	O	9.7	151	1de4	O	9.7	22
1eer	O	-	-	1fak	O	-	-
1fq1	O	-	-	1h1v	O	-	-
1ibr	O	-	-	2hmi	AB	9.7	14

* Complex category labels: E, Enzyme-Inhibitor or Enzyme-Substrate; A, Antibody-Antigen; O, Others; AB, Antigen-Bound Antibody.
